# Metagenomic Analysis Exploring Taxonomic and Functional Diversity of Soil Microbial Communities in Sugarcane Fields Applied with Organic Fertilizer

**DOI:** 10.1155/2020/9381506

**Published:** 2020-10-20

**Authors:** Ruoyu Li, Ziqin Pang, Yongmei Zhou, Nyumah Fallah, Chaohua Hu, Wenxiong Lin, Zhaonian Yuan

**Affiliations:** ^1^Key Laboratory of Sugarcane Biology and Genetic Breeding, Ministry of Agriculture, Fujian Agriculture and Forestry University, Fuzhou 350002, China; ^2^College of Agricultural, Fujian Agriculture and Forestry University, Fuzhou 350002, China; ^3^Genomics and Biotechnology, Fujian Agriculture and Forestry University, Fuzhou 350002, China; ^4^Province and Ministry Co-Sponsored Collaborative Innovation Center of Sugar Industry, Guangxi University, Nanning 530000, China

## Abstract

Organic fertilizers are critically important to soil fertility, microbial communities, and sustainable agricultural strategies. We compared the effect of two fertilizer groups (organic+chemical fertilizer: OM, chemical fertilizer: CK) on sugarcane growth, by observing the difference in microbial communities and functions, soil nutrient status, and agronomic characters of sugarcane. The results showed that the sugar content and yield of sugarcane increased significantly under organic fertilizer treatment. We believe that the increased soil nutrient status and soil microorganisms are the reasons for this phenomenon. In addition, redundancy analysis (RDA) shows that the soil nutrient condition has a major impact on the soil microbial community. In comparison with CK, the species richness of Acidobacteria, Proteobacteria, Chloroflexi, and Gemmatimonadetes as well as the functional abundance of nucleotide metabolism and energy metabolism increased significantly in the OM field. Moreover, compared with CK, genes related to the absorption and biosynthesis of sulfate were more prominent in OM. Therefore, consecutive organic fertilizer application could be an effective method in reference to sustainable production of sugarcane.

## 1. Introduction

Sugarcane (*Saccharum officinarum* L.; Poaceae) is the world's most important sugar and energy crop. Sugarcane plants are very tall, have a long production period, consume a lot of nutrients, and require a large amount of fertilizer as well as large amount of irrigation [1–3]. Balanced soil fertility plays a vital role in the growth of sugarcane. However, extensive use of chemical fertilizers severely depletes the nutrients in the soil, which will significantly reduce the yield of cultivated land [4]. Studies have shown that the application of organic fertilizer enhanced soil porosity, improved the soil aggregate structure, and adjusted various physical and chemical properties [4]. It is generally believed that a higher soil microbial diversity index is more conducive to improving the stability and resistance of soil ecosystems, ensuring normal operation of soil ecosystem function [5]. Organic fertilizers such as straw returning and pig manure contain a large number of microorganisms, and studies have shown that the nutrient matrix provided by the application of organic fertilizer eased competition among bacterial groups [6]. On the other hand, organic fertilizer itself contains a large number of microorganisms, substantially improving soil bacterial diversity [7]. The species and quantity of soil microorganisms not only are dynamic for the transformation and circulation of soil organic matter and soil nutrients but also act as reserve storage for the available nutrient of plants in the soil and are closely coupled with soil fertility. Soil may contain massive numbers of microbial species, such as fungi, viruses, bacteria, and archaea [8]. The majority of these taxa have not been described in detail and have unknown physiological and ecological attributes [9].

The concept of metagenomics was formally defined by Jo Handelsman of the University of Wisconsin. Metagenomics is a popular method of microbial research that uses high-throughput sequencing technology to characterize the taxonomic and functional attributes of biological communities [10]. Metagenomics avoids the separation of organisms and directly detects and quantifies DNA. It can quickly and accurately obtain abundant microbial data and has been widely used in the research of soil microorganisms [11–13]. Metagenomics has also been used in many sugarcane soil-related research projects. Studies have shown that no-tillage and bagasse mulching can affect the types and functions of soil microorganisms, and the impact on microbial function is less than that on community composition [14]. These functional changes may affect the productivity of sugarcane. Metagenomics has also been applied to detect sugarcane diseases. Studies have found that Sugarcane Yellow Canopy Syndrome is related to the function of specific soil microorganisms [15]. In addition, metagenomics has also found that earthworms can change the functional classification of soil microorganisms in a sugarcane field by increasing the accumulation of sugarcane biomass [16]. However, there are few reports on the relationship among the growth of sugarcane, the soil nutrients, and the changes of soil microorganisms under application of organic fertilizers. Thus, the objective of this study is to explore the effects of organic fertilizer on sugarcane growth, soil nutrients, and variation in the taxonomic composition of soil microbial communities.

## 2. Materials and Methods

### 2.1. Experimental Design and Sample Collection

The experimental field is situated in the Sugarcane Experimental Base (26°08′N, 119°23′E) of Fujian Agriculture and Forestry University, China. The area belongs to the subtropical monsoon climate region. The average annual temperature is 20°C, and the annual mean precipitation is 1363.91 mm. The soil was classified as yellow soil, and the maximum water holding capacity was 33.5%. Soil pH (5.21), soil organic matter (19.59 g kg^−1^), available phosphorus (16.1 mg/kg), and available potassium (89.47 mg/kg) were measured before the experiment. The sugarcane variety ROC22 [17] was selected as the experimental crop and planted in March 2012 at a seeding rate of about 100,000 double shoots/hm^2^ (1 hm^2^ = 10000 m^2^).

We used a randomized block design of two treatments: (1) conventional fertilization (CK), in which 300 kg/hm^2^ urea, 100 kg/hm^2^ K_2_O, and 400 kg/hm^2^ superphosphate were applied, and (2) chemical fertilizer combined with organic fertilizer (OM), in which 225 kg/hm^2^ urea, 75 kg/hm^2^ urea, 300 kg/hm^2^ superphosphate, and 1125 kg/hm^2^ organic fertilizer (organic content > 45%) were applied (Table 1). Each treatment consisted of 3 plots, with each plot containing 3 rows. The row spacing was 1.2 m, the row length was 8.0 m, and the total plot area was 28.8m^2^. 40% and 60% of the total fertilization amount was applied to the sugarcane at the seedling stage and the elongation stage, respectively. On March 8, 2019, soil samples were collected at the depth of approximately 10 cm of the topsoil; all soil samples were taken no less than two inches away from a plant stalk. Each soil sample was fully mixed to filter out impurities such as plant roots. A portion of each sample was air-dried to analyze physical and chemical properties of the soil, while the rest were stored at -80°C for DNA extraction. Sugarcanes were harvested on March 10, 2019, and each harvested plant was used to measure plant height, stem diameter, stem weight, hammer weight, and other indicators.

### 2.2. Measurement of Sucrose Content and Theoretical Yield

We measured the stalk height and diameter of 30 sugarcane plants that were randomly selected in each plot using a measuring tape and a Vernier caliper. In order to get the sucrose content, an Extech Portable Sucrose Brix Refractometer (Mid-State Instruments, CA, USA) was applied to measure the samples with the following formula [18]:

(1) $$\begin{array}{c} \text{sucrose}\left(\%\right)=\text{brix}\left(\%\right)\times 1.0825-7.703. \end{array}$$

For the estimation of theoretical production of sugarcane, these equations were followed: (1) Single stalk weight (kg) = [stalk diameter (cm)]^2^ × [stalk height (cm) − 30] × 1 (g/cm^3^) × 0.7854/1000 and (2) Theoretical production (kg/hm^2^) = single stalk weight (kg) × productive stem numbers (hm^2^).

### 2.3. Measurement of Soil Chemical Properties

The pH meter PHS-3C (INESA Scientific Instrument Co., Ltd., Shanghai, China) was used to estimate the soil pH [19]. Elemental analyzers (Thermo Scientific™, Waltham, MA, USA) were used to measure total soil sulfur (TS), total carbon (TC), and total nitrogen (TN) in extracts. Effective phosphorus (AP) was measured using hydrochloric acid and ammonium fluoride following the molybdenum blue protocol [20]. The alkaline hydrolyzable diffusion and potassium dichromate external heating methods were used to measure available nitrogen (AN) and organic matter (OM), respectively [21, 22]. We used ammonium acetate to extract available potassium (AK) and measured it by flame photometry [23]. Total potassium (TK) and total phosphorus (TP) were measured by first digesting the soil by adopting the H_2_SO_4_-HCLO_4_ method and then calculating the level as defined for AP and AK.

### 2.4. Soil DNA Extraction and Metagenomic Sequencing

Soil DNA was extracted using the E.Z.N.A.® DNA Kit (Omega Bio-Tek, Norcross, GA, U.S.) according to the manufacturer's instructions. Quality and concentration of extracted DNA were assayed with NanoDrop 2000.

The extracted DNA was fragmented into a size of approximately 300 bp with Covaris M220 (Gene Company Limited, China), and the TruSeq™ DNA Sample Prep Kit (Illumina, San Diego, CA, USA) was used to construct the paired-end library. Blunt-end fragments were ligated with adapters containing the full complement of sequencing primer hybridization sites. Paired-end sequencing was performed using the HiSeq 3000/4000 PE Cluster Kit and HiSeq 3000/4000 SBS Kit according to the manufacturer's instructions at Majorbio Bio-Pharm Technology Co., Ltd. (Shanghai, China).

### 2.5. Bioinformatics

**Figure 1:**
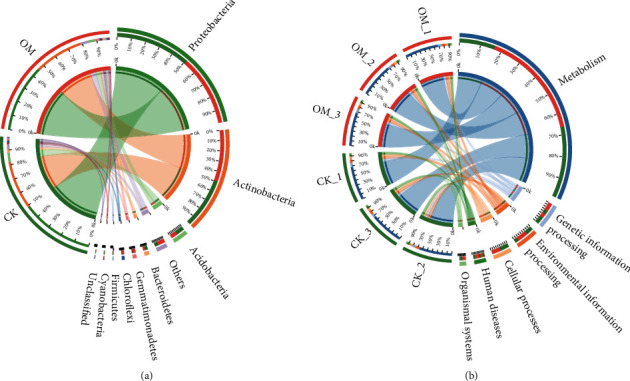
Circos diagram represents the microbial composition (a) and functional composition (b) of top phyla in two sugarcane fields.

SeqPrep V. 1.3.2 (https://github.com/jstjohn/SeqPrep) was used to strip adapter sequences. We used Sickle v. 1.33 (https://github.com/najoshi/sickle) to remove the low-quality reads (length < 50 bp or with a quality value < 20 or having N (ambiguity) bases). Metagenomic data were assembled using MEGAHIT v. 1.1.2 [24], and open reading frames (ORFs) of contigs in stitching results were predicted using MetaGene v. 2.20.0 [25]. The predicted ORFs with lengths over 100 bp were retrieved and translated into protein sequences using the NCBI translation table. CD-HIT v. 4.6.8 was used to cluster the predicted genes, and a nonredundant gene catalog was constructed using the longest sequences from each cluster [26]. Reads after quality control were mapped against the representative sequences using SOAPaligner v. 2.21 (R. [27]). Next, BLASTP v. 2.2.28+ [28] was used to align nonredundant gene sets to the NCBI NR database [29] for taxonomic annotation. BLASTP was also used for the Kyoto Encyclopedia of Genes and Genomes (KEGG) annotation [30]. The aligned sequence data were fed to KOBAS v. 2.0 for functional annotation at the three levels of L1, L2, and L3 [31]. Based on the annotation results, Circos v. 0.67-7 [32] was used to present the corresponding components of microorganisms and KEGG functional annotation in two groups (Figure 1). Differences in microbial taxa between OM and CK were calculated and visualized using linear discriminant analysis effect size (LEfSe), and LDA scores exceeding 3.5 were confirmed by LEfSe [33]. R v. 3.5.1 (https://www.r-project.org/) was used for PCoA analysis and mapping. PCoA analysis was performed to find the most essential elements and structures in the data. Analysis of similarities (ANOSIM) was used to test whether the difference between groups was significantly greater than that within groups, so as to judge whether grouping was meaningful. Redundancy analysis (RDA) was executed in R to analyze the relationship between dominant taxa of microorganisms and soil properties.

## 3. Results

### 3.1. Soil Physiochemical Properties

The results showed that the application of organic fertilizers affected sugarcane physicochemical properties. The pH of the soil increased significantly (*p* < 0.05), which effectively relieved the acidification of the soil. Furthermore, soil organic matter content increased significantly, with an increase amplitude of 98.0% (*p* < 0.05). Soil TS contents of the OM treatment were significantly lower compared to the CK treatment (*p* < 0.05). Soil TN, TP, C_N, AN, and AP increased slightly after applying organic fertilizer, but the increases were not significant (Table 2).

### 3.2. Sugarcane Agronomic Properties and Production

The results revealed that the application of organic fertilizers affected some sugarcane agronomic parameters significantly. The brix of sugarcane increased from 8.17% to 11.16%, with an increase amplitude of 36.6% (*p* < 0.05). The production of sugarcane increased from 38,920.82 kg/hm^2^ to 54,367.45 kg/hm^2^, with an increase amplitude of 39.7%. The stalk height, stalk diameter, and productive stem numbers of sugarcane increased slightly, but the increase amplitude was not significant (Table 3).

### 3.3. Principal Coordinate Analysis (PCoA) of Soil Microorganisms

**Figure 2:**
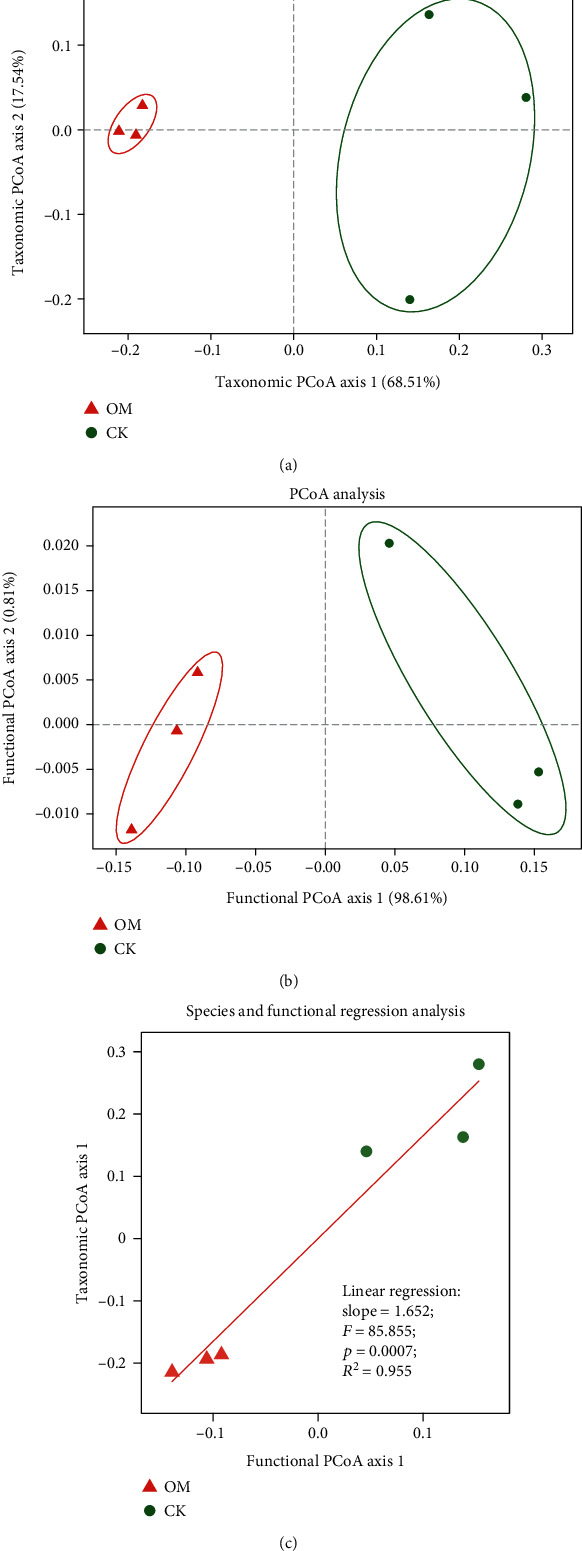
Effects of experimental treatments on the taxonomic (a) and functional (b) composition of soil microbial communities and the relationship between the taxonomic and functional compositions (c). ANOSIM indicates the significance of the difference between groups.

We found that the composition and function of the rhizosphere soil microbiota differed with respect to fertilizer regime. Unconstrained principal coordinate analysis (PCoA) of Bray-Curtis distances revealed that the rhizosphere microbiota of both OM and CK treatments formed two distinct clusters, which separated along the first coordinate axis. The principal coordinate axis 1 (PC1) and the principal coordinate axis 2 (PC2) can be used to interpret 68.51% and 17.54% of the variation in taxonomic classification (Figure 2(a)) and 98.61% and 0.81% in functional capacity (Figure 2(b)). The cumulative variance contribution rates of the two principal components reached 86.05% and 99.42% in classification and function, respectively, which can separate the two groups based on their specific characteristics. The ANOSIM analysis confirmed that the soil microbial composition and function were significantly different under OM and CK treatments (*p* < 0.05). Therefore, on the foundation of applying chemical fertilizer, we can conclude that organic fertilizer is the main factor affecting the taxonomic composition and function of soil microbial communities. In addition, the correlation analysis of the principal component coordinate axis of microbial species exhibits a significant linear relationship between community composition and function (Figure 2(c), *R*^2^ = 0.955, *p* < 0.01).

### 3.4. Microbial Community Analysis

**Figure 3:**
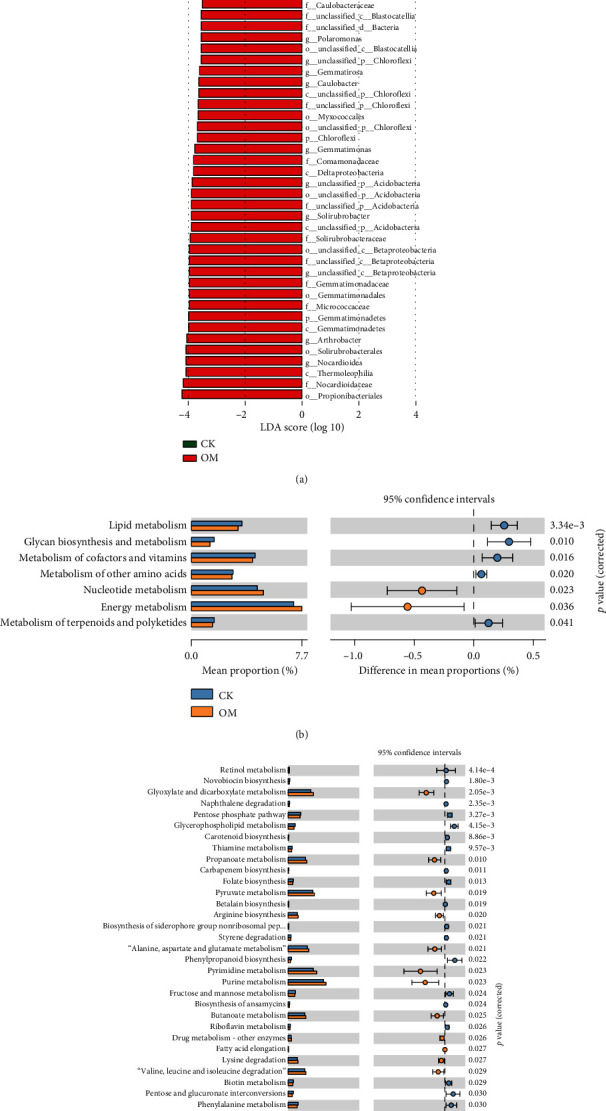
Histograms showing the phylogenetic distribution of the microorganism lineages associated with the two sugarcane fields. Lineages with LDA values higher than 3.5 are displayed (a). Different-colored regions represent different constituents (red: OM, green: CK). Extended error bar plots indicate significantly different predicted functional categories at level 2 (b) and level 3 (c) detected in CK and OM treatments (*p* < 0.05, mean proportion, *n* = 3).

A total of 13,101 microorganism species were detected in the OM treatment, of which bacteria accounted for 98.61% and other microorganisms accounted for 1.39%. In the CK treatment, 13,720 species were detected and the proportion of bacteria and other microorganisms were 99.24% and 0.76%, respectively. In both fields, the top dominant microorganism phyla were identified, namely, Proteobacteria, Actinobacteria, Bacteroidetes, Gemmatimonadetes, Chloroflexi, Firmicutes, and Cyanobacteria (Figure 1(a)). The dominant phyla in the microbial communities showed great differences between the two fields. The OM field was significantly enriched with Acidobacteria, Proteobacteria, Chloroflexi, and Gemmatimonadetes compared with the CK field. However, at the genus level, the OM field was more enriched with *Pyrinomonas*, *Solirubrobacter*, *Arthrobacter*, *Nocardia*, *Gemmatimonas*, *Polaromonas*, and *Caulobacter* than the CK field (Figure 3(a)).

### 3.5. Relationship between Microbial Community Structure and Environmental Characteristics

**Figure 4:**
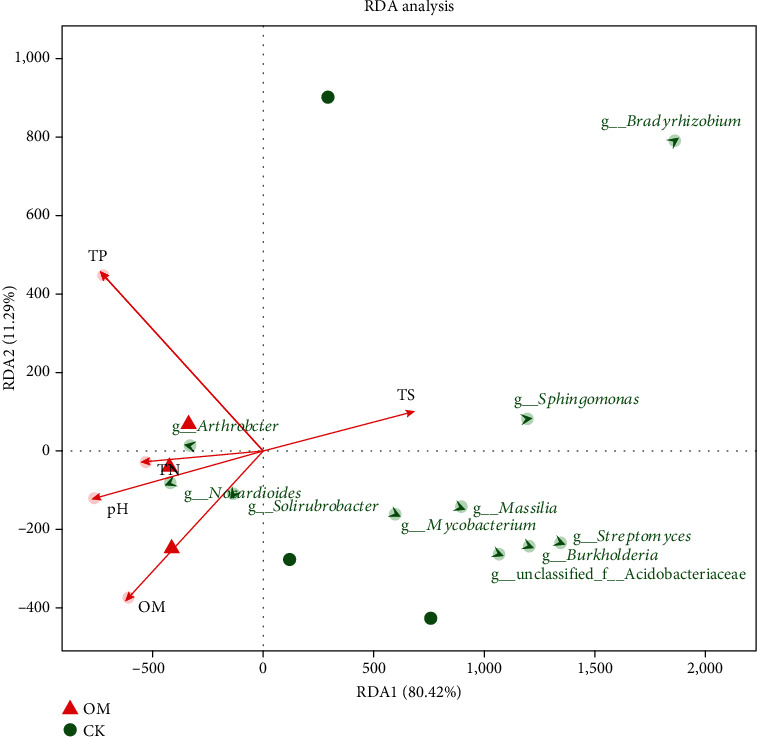
Redundancy analysis of soil properties and dominant microorganism genus. Soil property abbreviations: TN: total nitrogen; TP: total phosphorus; TS: total sulfur; OM: organic matter.

Redundancy analysis (RDA) was used to assess the environmental factors influencing the microbial structure. The results revealed that the microbial community structure was affected by primary environmental characteristics. The RDA results suggested that soil pH, OM, TN, TP, and TS accounted for 91.71% of the total shift in microbial communities (Figure 4). The OM samples were completely separated from the CK samples. *Bradyrhizobium*, *Sphingomonas*, *Massilia*, *Mycobacterium*, *Streptomyces*, and *Burkholderia* were positively associated with TS but negatively correlated with TN, TP, OM, and pH. In addition, *Solirubrobacter*, *Arthrobacter*, and *Nocardioides* were positively correlated with pH, TP, TN, and OM, but negatively correlated with TS (Figure 4).

### 3.6. Microorganism Function Analysis

According to the aligned results of KEGG, the total functions (6, 49, and 409) at the three levels (L1, L2, and L3) were identified from the microorganisms obtained from two soil samples. The majority of sequences were functionally associated with metabolism (70.24%-70.64%), genetic information processing (8.00%-8.41%), environmental information processing (7.47%-7.81%), cellular processes (6.25%-6.76%), human diseases (4.37%-4.41%), and organismal systems (2.78%-2.78%) (Figure 1(b)). We also observed differences in seven classified functions at the L2 level through statistical analysis. Application of OM significantly increased the relative abundance of nucleotide metabolism and energy metabolism compared with CK treatment (Figure 3(b)). However, compared with CK, OM decreased in seven functional categories, including lipid metabolism, glycan biosynthesis and metabolism, metabolism of cofactors and vitamins, metabolism of other amino acids, and metabolism of terpenoids and polyketides (Figure 3(c)).

### 3.7. Increased Sulfur Metabolism to Biomass

**Figure 5:**
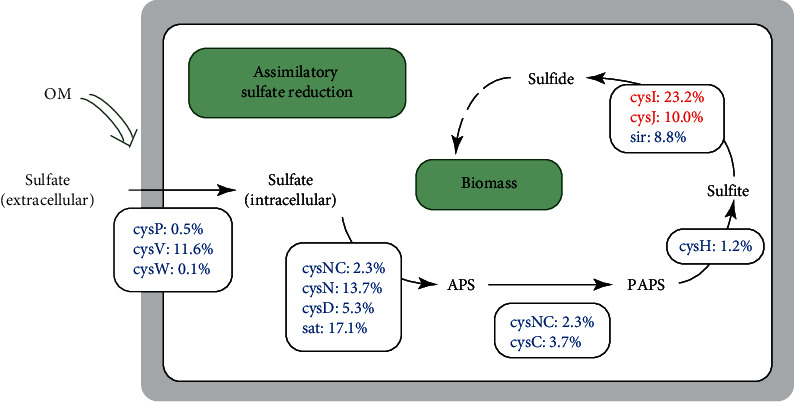
The pathway of import and assimilatory sulfate reduction under organic fertilizer. Percentages indicate the relative variation compared with CK. Percentages given in red and blue represent an increase or decrease in the abundance of the genes.

Sulfur metabolism was significantly enriched in OM compared to CK, as displayed by the KEGG results (Figure 3(c)). Plants absorb sulfate through the root system to maintain a normal development. Sulfate is reduced by an assimilation sulfate reduction pathway and assimilated into structural and functional organic sulfides. In this study, we explored the promotion of sugarcane biomass by soil microorganisms based on the assimilation sulfate reduction pathway. The abundance of the three sulfate import genes (*cysP*, *cysV,* and *cysW*) increased by 0.5%, 11.6%, and 0.1% in OM compared to the CK group, respectively. Most of the genes in the assimilation sulfate reduction pathway were also more abundant in the OM data set, such as sulfate adenyitransferase (*cysNC*, *cysN*, *cysD*, and *sat*), adenylyl-sulfate kinase (*cysNC*, *cysC*), phosphoadenylyl-sulfate reductase (*cysH*), and assimilatory sulfite reductase (*sir*). Only assimilatory sulfite reductase (*cysI*, *cysJ*) showed a decline (Figure 5). The conclusions are consistent with the result of Zhang et al. [34], whose results suggested that the application of organic fertilizer could promote the absorption of soil sulfate by stimulating the growth of soil microorganisms.

## 4. Discussion

Our research explored the impact of organic fertilizer application on soil physiochemical properties, agronomic traits, and microbial composition in the sugarcane cropping system. The results revealed that the application of organic fertilizer improved the sugarcane agronomic traits and yield, a finding that is with previous studies on strawberry [35], rice [36], and watermelon [37]. In addition, the application of organic fertilizers can also increase soil nutrient status and reduce soil acidification in sugarcane planting systems [38–40]. Based on the results of our study, we therefore believe that mitigating soil acidification and optimizing the soil nutrient status of sugarcane fields can help improve sugarcane yields.

The pH value and N, P, and S contents of soil are very sensitive to the changes of organic matter and can be used as indicators to evaluate soil quality. Moreover, these soil indicators can influence the composition of soil microorganism communities. Although the overall number of soil microbial species remained almost the same, the dominant species changed significantly. This finding reflects those of previous studies, in which the application of organic fertilizer changed the characteristics of soil microbial communities [40–42]. The effects of organic fertilizers on soil microorganisms have been widely investigated in various agricultural systems [43–45]. Our RDA analysis revealed that the changes in soil microbial community composition were associated with soil physiochemical properties. In particular, OM treatment has a significant influence on microbial community composition. In the OM treatment, the phyla Acidobacteria, Proteobacteria, Chloroflexi, and Gemmatimonadetes were abundant compared with the CK community. Members associated with Acidobacteria generally accounted for 20% to 50% of the total amount of bacteria. Acidobacteria has been reported to have a variety of functions, such as the degradation of plant polymers [46], participation in the iron cycle [47], photosynthesis capability [48], and participation in the carbon cycle [49]. Proteobacteria is a phylum with significant functions in the degradation of biofertilizers [50] as well as in sulfate reduction [51]. Chloroflexi is a kind of photoautotrophic bacteria with the ability to degrade organic matter, and they tend to grow in a nutrient-rich environment [52]. Gemmatimonadetes reproduce by germination, and its members have salt-resistant properties. Results of previous studies have shown that Gemmatimonadetes is positively correlated with soil conductivity [53]. The OM field was also characterized by *Pyrinomonas*, *Solirubrobacter*, *Arthrobacter*, *Nocardia*, *Gemmatimonas*, *Polaromonas*, and *Caulobacter*, unlike the CK field. Among them, *Pyrinomonas* belongs to Acidobacteria; *Solirubrobacter*, *Arthrobacter*, and *Nocardia* belong to Actinobacteria; *Gemmatimonas* belong to Gemmatimonadetes; and *Polaromonas* and *Caulobacter* belong to Proteobacteria. These genera were seldom reported in previous studies in fields cultivated with sugarcane. However, the phyla of these genera have also been reported many times in other sugarcane soil studies. Acidobacteria and Actinomycetes are also more common in sugarcane soils treated with nitrogen fertilizer [54, 55]. Proteobacteria significantly increased in bagasse mulching sugarcane soil [14]. In comparison with CK, two functional classes, namely, nucleotide metabolism and energy metabolism, increased significantly in the OM field. In addition, we analyzed the effects of soil microorganisms on sulfur metabolism and found that the sulfate import genes such as *cysP*, *cysV*, and *cysW*, and most of the genes involved in the sulfate reduction pathways, increased in the OM. This finding is consistent with previous studies and suggests that the application of organic fertilizer can promote the conversion process of extracellular sulfate to biomass in sulfur metabolism [34, 56].

In summary, we assess the effects of organic fertilizer on the microbial community of sugarcane soil using a metagenomic approach and found that there was a close relationship between the parameters of sugarcane, soil nutrients, and soil microorganisms. Our study provides insights into reforming cropping soil and increasing the yield of sugarcane and other plants in the future.

## Figures and Tables

**Table 1:** Soil management and fertilizer regimes every year.

Sugarcane fields	Soil fertilizer regimes
CK	Fertilization with potassium oxide (100 kg/hm^2^), calcium superphosphate fertilizer (400 kg/hm^2^), and urea (300 kg/hm^2^) in April every year.
OM	Fertilization with potassium oxide (75 kg/hm^2^), calcium superphosphate fertilizer (300 kg/hm^2^), and urea (225 kg/hm^2^) in April every year. Fertilization with organic fertilizer (1125 kg/hm^2^) (26.4% organic C, 2.5% total N, 1.6% P_2_O_5_, and 1.3% K_2_O, made of composted rice straw and pig manure by Tianniang Ltd. of Changshu, China) in April every year.

**Table 2:** Impact of organic fertilizer application on soil physiochemical properties of sugarcane.

Specimen name	CK	OM
pH	4.78 ± 0.23^b^	6.48 ± 0.12^a^
OM	15.42 ± 4.43^b^	30.53 ± 6.15^a^
TN	0.90 ± 0.21^a^	1.24 ± 0.41^a^
TP	0.54 ± 0.18^a^	0.79 ± 0.09^a^
TK	23.57 ± 1.45^a^	25.38 ± 0.42^a^
TS	0.21 ± 0.03^a^	0.12 ± 0.02^b^
C_N	18.10 ± 8.20^a^	26.34 ± 10.58^a^
AN	67.78 ± 9.67^a^	70.10 ± 2.79^a^
AK	78.36 ± 8.95^a^	89.60 ± 7.54^a^
AP	15.38 ± 5.80^a^	14.89 ± 1.76^a^

^a^All values are the mean of three replicates. Numbers followed by “±” represent the standard errors. ^b^The data with identical superscript letters indicate that the mean values are not significantly different.

**Table 3:** Impact of organic fertilizer application on sucrose content, growth parameters, and yield of sugarcane.

Treatment	CK	OM
Stalk height (cm)	236.67 ± 15.28^a^	258.67 ± 10.26^a^
Stalk diameter (cm)	1.63 ± 0.07^a^	1.81 ± 0.12^a^
Sucrose content (%)	8.17 ± 1.47^b^	11.16 ± 0.37^a^
Available stalk number (hm^−2^)	89,556 ± 4,018^a^	92,000 ± 8,664^a^
Production (kg/hm^2^)	38,921 ± 5,592^b^	54,367 ± 6,143^a^

^a^All values are the mean of three replicates. Numbers followed by “±” represent the standard errors. ^b^The data with identical superscript letters indicate that the mean values are not significantly different.

## Data Availability

The sequencing reads were deposited in the Sequence Reads Archive database of the National Center for Biotechnology (accession no. SRA: SRP280068).
